# Synergetic delivery of artesunate and isosorbide 5-mononitrate with reduction-sensitive polymer nanoparticles for ovarian cancer chemotherapy

**DOI:** 10.1186/s12951-022-01676-3

**Published:** 2022-11-05

**Authors:** Guang Li, Mingjian Ling, Kunyi Yu, Wei Yang, Qiwen Liu, Lijuan He, Xuzi Cai, Min Zhong, Ziyi Mai, Rui Sun, Yuanling Xiao, Zhiqiang Yu, Xuefeng Wang

**Affiliations:** 1 Department of Obstetrics and Gynecology, The Third Affiliated Hospital of Southern Medical University, Guangzhou, 510630 China; 2 Southern Medical University Shenzhen Stomatology Hospital (Pingshan), Shenzhen, 518000 China; 3 Shenzhen Longhua District Central Hospital, Shenzhen, 518110 China; 4 Zhujiang Hospital of Southern Medical University, Guangzhou, 510280 China; 5 School of Pharmaceutical Sciences, Guangdong Provincial Key Laboratory of New Drug Screening, Southern Medical University, Guangzhou, 510515 China; 6 Department of Gynecology, Obstetrics and Gynecology Center, Zhujiang Hospital, Southern Medical University, Guangzhou, 510280 China; 7 Department of Laboratory Medicine, Dongguan Institute of Clinical Cancer Research, Affiliated Dongguan Hospital, Southern Medical University, Dongguan, 523018 China

**Keywords:** Artesunate, Isosorbide 5-mononitrate, DNA damage, Mitochondrial damage, Cell cycle arrest, Ovarian cancer

## Abstract

Ovarian cancer is a highly fatal gynecologic malignancy worldwide. Chemotherapy remains the primary modality both for primary and maintenance treatments of ovarian cancer. However, the progress in developing chemotherapeutic agents for ovarian cancer has been slow in the past 20 years. Thus, new and effective chemotherapeutic drugs are urgently needed for ovarian cancer treatment. A reduction-responsive synergetic delivery strategy (PSSP@ART-ISMN) with co-delivery of artesunate and isosorbide 5-mononitrate was investigated in this research study. PSSP@ART-ISMN had various effects on tumor cells, such as (i) inducing the production of reactive oxygen species (ROS), which contributes to mitochondrial damage; (ii) providing nitric oxide and ROS for the tumor cells, which further react to generate highly toxic reactive nitrogen species (RNS) and cause DNA damage; and (iii) arresting cell cycle at the G0/G1 phase and inducing apoptosis. PSSP@ART-ISMN also demonstrated excellent antitumor activity with good biocompatibility in vivo. Taken together, the results of this work provide a potential delivery strategy for chemotherapy in ovarian cancer.

**Graphical Abstract:**

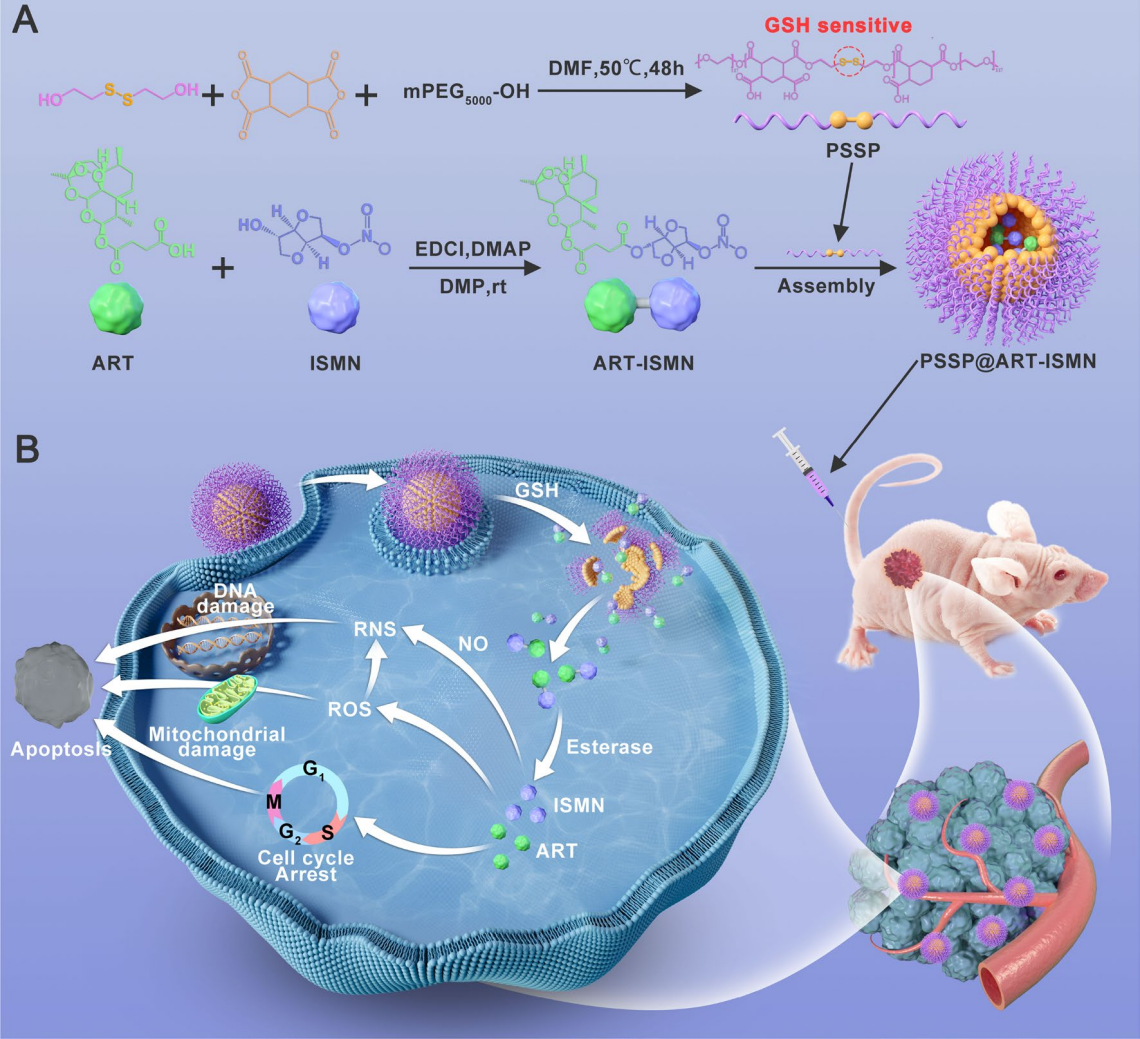

**Supplementary Information:**

The online version contains supplementary material available at 10.1186/s12951-022-01676-3.

## Introduction

Ovarian cancer is a highly fatal gynecologic malignancy with limited treatment options worldwide [1, 2]. Approximately 70% of patients are in the advanced stage of the disease once diagnosed [3]. Unfortunately, limited improvement has been achieved in the last 20 years, making it urgent to find new therapeutic strategies. Chemotherapy is widely used in the treatment of ovarian cancer as an initial and maintenance therapy strategy [4]. However, the therapeutic efficacy of chemotherapy drugs is often compromised by drug resistance and systemic side effects, leading to physical pain and inefficient treatment [5]. Thus, the search for new chemotherapeutic agents is desperately needed.

The active ingredients in traditional Chinese medicines are becoming effective drugs in the treatment of malignancies and shedding light on ovarian cancer therapy [6]. Artesunate (ART) is a naturally occurring sesquiterpene lactone extracted from the traditional Chinese medicine *artemisia annua* [7–9], which has been demonstrated to be effective against malaria [10–12]. Recent studies have found that ART also exhibits extensive antitumor effects in liver cancer [13–15], oral tongue squamous cell carcinoma [16], bladder cancer [17, 18], and breast cancer [19–21]. However, challenges, such as poor solubility, high toxicity, and off-target accumulation, have limited its clinical use as a free ART in monotherapy [21, 22].

Nitric oxide (NO) gas therapy is emerging as a promising therapeutic strategy for malignant tumors. The combination of NO gas therapy and chemotherapy has been shown to have synergistic therapeutic effects [23]. Previous studies have reported that a high level of NO inhibits proliferation and sensitizes tumors to chemotherapeutic drugs [23], making it an ideal candidate for chemosensitizers. Isosorbide 5-mononitrate (ISMN) is an NO donor mainly used in the treatment of angina pectoris [24–26], which has attracted the attention of many researchers due to its potential application in the field of cancer therapy [27, 28]. The NO released by ISMN can react with reactive oxygen species (ROS) to produce a highly toxic RNS, which has been considered a far-sighted strategy for cancer treatment [21, 29, 30].

The present study developed a glutathione (GSH)-responsive nanoparticles (NPs) loaded with ART and ISMN (PSSP@ART-ISMN) (Graphical abstract A). The disulfide bond (S–S) in the polymer shell can be reduced to the hydrophilic thiol group by GSH, which facilitates the release of ART and ISMN (Graphical abstract B). The further mechanistic study revealed that ART elevated the intracellular levels of ROS, induced the formation of RNS, and arrested the cell cycle at the G0/G1 phase, thereby leading to cell apoptosis. Moreover, the PSSP@ART-ISMN exhibited excellent antitumor efficacy and negligible side effects in vivo. Collectively, these characteristics make it a desirable candidate for ovarian cancer chemotherapy.

## Materials and methods

### Materials and measurements

ART and ISMN were purchased from Energy Chemical (Shanghai, China), mPEG5000 was sourced from AVT Pharmaceutical Tech Co., Ltd (Shanghai, China), apoptosis detection kit, ROS assay kit, and NO fluorescence probe were obtained from Beyotime (Shanghai, China), and RNS fluorescence probe was provided by BestBio (Nanjing, China). The polyclonal antibody was bought from Bioss (Beijing, China). Cell culture vessels were purchased from Nest Biotechnology (Wuxi, China).

^1^H NMR spectra were recorded on a 400 MHz NMR spectrometer (Bruker, Switzerland). NP size and zeta potential were obtained via dynamic light scattering (DLS) analysis (Malvern, UK). The morphology and diameter were characterized using a transmission electron microscope (TEM). Drug loading rates for different NP formulations were measured using the ultraviolet (UV)-vis spectrum (Metash, UV-5500PC, China). The stability of PSSP@ART-ISMN under physiological conditions was investigated in 10% fetal bovine serum (Excell Bio, China) over the course of seven days using DLS.

### Synthesis of ART-ISMN prodrug and PSSP

ART (384 mg), EDC (288 mg), and DMAP (12.2 mg) were dissolved in dry dichloromethane (4 mL), the mixture was stirred in an ice bath for 30 min, and the solution was stirred at room temperature overnight after addition of ISMN (286.5 mg). The ligation reaction was stopped by adding water (10 mL), and the crude products were filtered and dried in a rotary steamer and purified via silica gel flash column chromatography (EA/PE = 2:1) to yield the desired compound. PSSP was synthesized based on our previous work [21, 29, 30]. Detailed synthetic routes are described in the supporting information section (Additional file 1: Schemes S1, S2).

#### ART-ISMN

^1^H NMR (400 MHz, CDCl3) δ 5.77 (d, J = 9.8 Hz, 1H), 5.43 (s, 1H), 5.35 (d, J = 2.8 Hz, 1H), 5.23 (d, J = 2.3 Hz, 1H), 4.99 (s, 1H), 4.50 (d, J = 4.8 Hz, 1H), 4.14–3.95 (m, 3H), 3.90 (dd, J = 11.2, 5.5 Hz, 1H), 2.81–2.69 (m, 2H), 2.65 (dd, J = 12.7, 6.6 Hz, 2H), 2.59–2.48 (m, 1H), 2.35 (dd, J = 13.9, 3.8 Hz, 1H), 2.02 (d, J = 12.5 Hz, 1H), 1.89 (dd, J = 8.8, 4.9 Hz, 1H), 1.61 (d, J = 10.7 Hz, 4H), 1.52–1.45 (m, 1H), 1.43 (s, 3H), 1.43 (s, 2H), 1.32–1.21 (m, 3H), 0.96 (d, J = 5.8 Hz, 3H), 0.84 (d, J = 7.1 Hz, 3H).

#### PSSP

^1^H NMR (400 MHz, DMSO) δ 12.24 (s, 112H), 4.30–4.09 (m, 281H), 3.52 (s, 825H), 2.91 (s, 131H), 2.80 (d, *J* = 6.3 Hz, 125H), 2.34 (s, 81H), 2.09–1.97 (m, 77H).

### Preparation and characterization of PSSP@ART-ISMN

First, 10 mg of PSSP and 2 mg of ART-ISMN were dissolved in 0.8 mL of DMF. The mixture was stirred for 30 min and PSSP@ART-ISMN was obtained via drop-wise precipitation in deionized water (2.4 mL). The obtained PSSP@ART-ISMN was purified by dialysis (MW = 3500 Da) to remove the non-encapsulated drugs.

### Drug release studies

The GSH responsiveness of nanoparticles was measured by adding 10 mM GSH for 1 h. The morphology and particle size of nanoparticles after response were measured by transmission electron microscopy and DLS, respectively. A total of 2 mL of PSSP@ART-ISMN (2 mg/mL) was placed in a dialysis bag, which was immersed in 18 mL of phosphate-buffered saline (PBS, pH = 6.5 and pH = 7.4) and 10 mM GSH. At the given times, the withdrawn external solution (0.2 mL) was replenished with the same volume of fresh medium. The cumulative amount of ART-ISMN released in vitro was determined using UV–vis spectra.

### Cell lines and animals

SKOV3, HO8910, and IOSE-80 cells (Wuhan Sunncell Biotechnology Co., Ltd, Chian) were cultured in RPMI-1640 medium (Gibco) containing 10% FBS (HAKATA, China) and 1% penicillin/streptomycin. BALB/c nude mice (20 g) were obtained from Guangdong Medical Laboratory Animal Center.

### Cellular uptake

SKOV3 cells were cultured overnight on 6-well plates (5 × 10^5^/well). After incubated with PSSP@Rh B for 2, 4, and 6 h, the nucleus of SKOV3 were visualized by DAPI (Beijing Solarbio Science & Technology Co., Ltd, China). Then, the cells were observed using confocal laser scanning microscopy (CLSM). To perform flow cytometry, the cells were collected for the intracellular uptake analysis after being treated with PSSP@Rh B for 2, 4, and 6 h, respectively.

### Cell viability studies

Two ovarian cancer cell types (SKOV3, HO8910) and a normal ovarian epithelial cell line (IOSE-80) were cultured on 96-well plates (5 × 10^3^/well) overnight. Since PSSP was demonstrated to be safe in previous work [6, 31], it was not necessary to test their biocompatibility in the present study. The cells were treated with PSSP@ART-ISMN, PSSP@ART, ART-ISMN, ART, and PBS at ART concentrations ranging from 0.3 µM to 40.0 µM. After incubating for 72 h, the cellular viability was assessed via an methyl thiazolyl tetrazolium (MTT) colorimetric assay.

### Apoptosis analysis

SKOV3 cells (8 × 10^3^/well) were cultured on 12-well plates overnight. After incubation with PSSP@ART-ISMN, PSSP@ART, ART-ISMN, ART, and PBS for 48 h (ART: 5 µM, ART-ISMN: 5 µM), the cells were collected for double staining with FITC/PI (Yeason, China) according to the manufacturer’s instructions.

### Intracellular ROS, NO, and RNS release

The intracellular NO generation was detected by DAF-FM DA (Beyotime, S0019). The intracellular levels of ROS and RNS were measured by DCFH-DA (Beyotime, S0033S) and O52D (BestBio, BB-460567). SKOV3 cells (1 × 10^5^/well) were incubated on 12-well plates overnight, followed by incubation with PBS, ART, ART-ISMN, PSSP@ART, and PSSP@ART-ISMN for 48 h (ART: 5 µM, ART-ISMN: 5 µM). The cells were then washed with PBS three times, harvested, and incubated with a fluorescence probe (DAF-FM DA 1:1,000 dilutions, DCFH-DA 1:800 dilutions, and O52D fluorescent probe 1:100 dilutions) for 30 min. Finally, the cells were rinsed with PBS thrice before imaging, and quantitative analysis was performed using flow cytometry.

### Cell cycle studies

SKOV3 cells were incubated on six-well plates (1.5 × 10^5^/well) with serum-free media to synchronize the cell cycle at the G0/G1 stage. After treatment with different agents for 48 h (ART: 5 µM, ART-ISMN: 5 µM). Cell cycle analysis was performed using propidium iodide (PI) staining and analyzed via flow cytometry. Cell population percentages were calculated using ModFit LT 5 software (Verity Software House, Topsham, ME, USA).

### Tissue distribution

SKOV3 cell transplantation model was established in female nude mice. Cy5.5-loaded PSSP was prepared to assess the bio-distribution of PSSP@ART-ISMN. An IVIS Spectrum live animal imaging system (IVIS Lumina, USA) was used to capture the fluorescence signal 0, 2, 4, 8, 12, 24, and 48 h post-injection (E_ex_ = 640 nm, E_em_ = 670 nm). Mice were sacrificed at the end of the experiments, and tumors and major tissues were resected for in vitro fluorescence imaging and histological staining.

### Western blotting

SKOV3 cells were cultured on 6-well plates overnight, after being treated with ART, ART-ISMN, PSSP@ART, and PSSP@ART-ISMN for 48 h (ART: 5 µM, ART-ISMN: 5 µM), the total proteins were collected from SKOV3 cells and quantified by a BCA protein assay kit. Then equal quantities of these proteins were loaded on 10% SDS-PAGE (GenScript) and subjected to polyvinylidene difluoride (PVDF) membrane. Then, the membranes were blocked with 5% non-fat milk for 2 h and followed by overnight incubation with primary antibodies (Cyclin D,γ-H2A.X, Caspase-3, Bak, Bcl2, P53, Cytochrome C, and β-actin). Then the secondary IgG antibody was applied and incubated at RT for 2 h. Finally, each band was visualized using an image analysis system (Protein Simple, USA).

### Therapeutic effect and systemic toxicity in vivo

The SKOV3 tumor-bearing mice were used to evaluate the therapeutic effect and safety of NPs. The tumor volume (mm^3^) and body weight were measured daily. The mice were sacrificed at the end of the treatment, and tumors and major organs were harvested for H&E and immunofluorescence staining, which was performed by Servicebio Biological Technology.

## Results and discussion

### Synthesis and characterization of PSSP@ART-ISMN

**Fig. 1:**
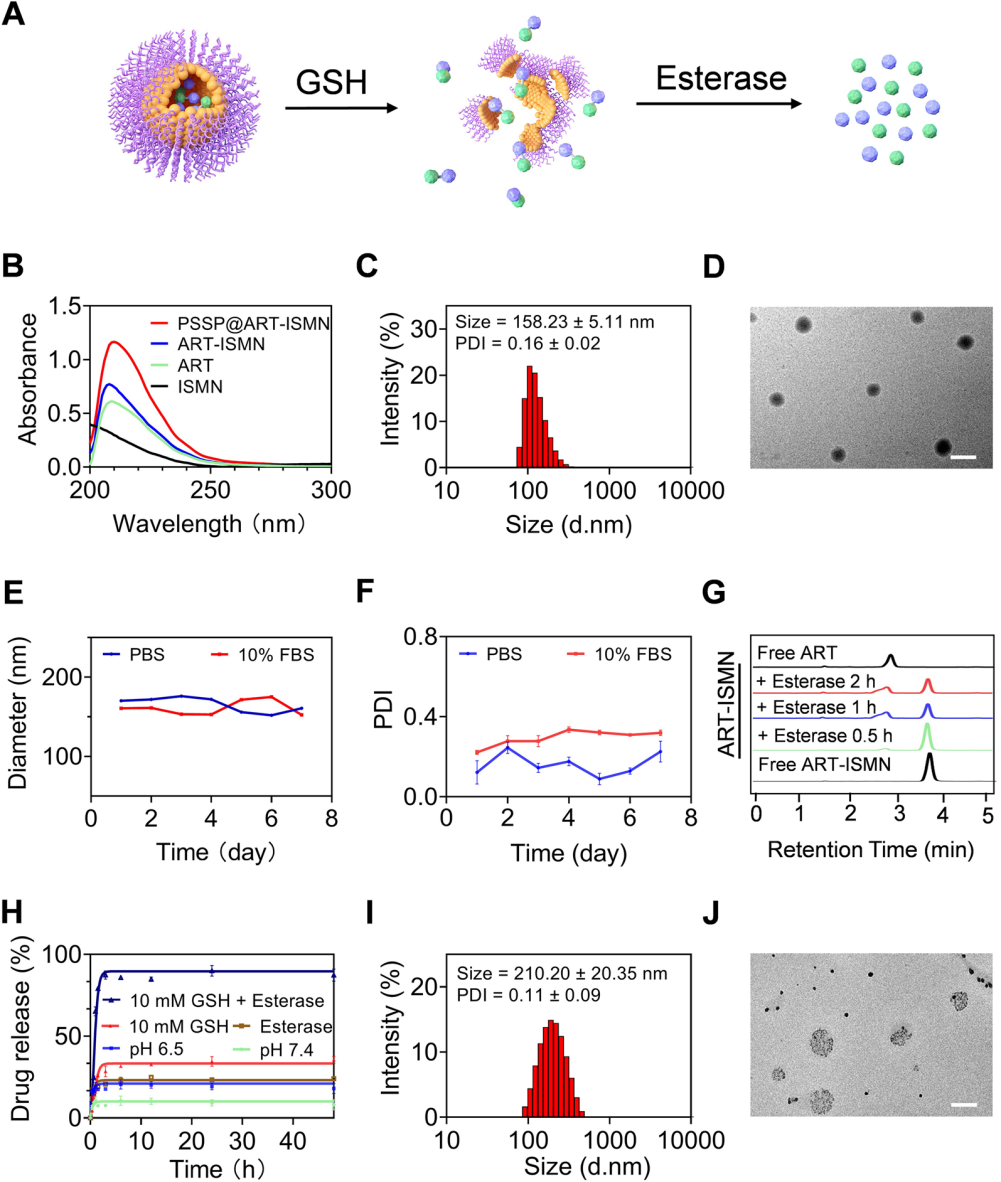
Characterization of PSSP@ART-ISMN. **A** Schematic diagram of PSSP@ART-ISMN decomposition process. **B** UV–vis spectra of ART, ISMN, ART-ISMN, and PSSP@ART-ISMN. **C**, **D** DLS data and representative TEM images of PSSP@ART-ISMN, scale bar = 200 nm. Diameter (**E**) and PDI (**F**) of PSSP@ART-ISMN in PBS with or without 10% FBS. **G** HPLC spectra of ART-ISMN incubated in PBS containing esterase. **H** Cumulative ART release profiles for PBS (pH = 7.4), PBS (pH = 6.5), and in the presence of GSH (10 mM) with or without esterase (30 U/mL). DLS data (**I**) and representative TEM images **J** of PSSP@ART-ISMN with 10 mM GSH

The PSSP and ART-ISMN were designed and synthesized using the process represented in Additional file 1: Schemes S1 and S2. Representative ^1^H NMR spectra are shown in Additional file 1: Figures S1 and S2, confirming the successful synthesis of PSSP and ART-ISMN. The ART was linked to ISMN via an ester bond, which was degraded in tumor milieus, resulting in synchronous release of ART and ISMN (Fig. 1A). PSSP and ART-ISMN were assembled into NPs using the nano-precipitation method. After the optimization of the ART-ISMN and PSSP formulation and assembly process, the smallest-sized NPs with moderate polydispersity and the highest drug loading rate (DL%) were obtained at a mass ratio of 2:10 (Additional file 1: Table S1), which was selected as the optimized ratio for subsequent research. The absorption spectra of PSSP@ART-ISMN indicated that ISMN was successfully encapsulated in the amphiphilic polymer PSSP (Fig. 1B). The drug loading rate and encapsulation efficiency (EE%) of PSSP@ART-ISNM were 3.97% and 24.90%, respectively. DLS results showed that the particle size of PSSP@ART-ISMN was 158.23 ± 5.11 nm, and its polydispersity indexes (PDI) was 0.16 (Fig. 1C). TEM images showed that the obtained representative NPs exhibited uniform spherical morphologies with a 160-nm diameter (Fig. 1D). The above results indicate that PSSP@ART-ISMN has a uniform particle size and distribution. The zeta potential of PSSP@ART-ISMN was about − 24.97 mV, suggesting excellent physical stability in blood circulation. In addition, the size and PDI remained constant after storage in PBS and 10% FBS for seven days (Fig. 1E, F), indicating the stability of long circulation.

High-performance liquid chromatography (HPLC) analysis was performed to test the responsiveness of ART-ISMN. The appearance of the ART peak indicates that free ART can be released from ART-ISMN (Fig. 1G). PSSP@ART-ISMN drug release profiles were then measured at pH 7.4, and pH 6.5, and in the presence of GSH (10 mM) with or without esterase (30 U/mL). As anticipated, there was an increased release of 10 mM GSH with esterase, with over 80% of drug released within 3 h (Fig. 1H), indicating that NPs were rapidly released in the tumor microenvironment with a high GSH content. The DLS data and TEM images showed that the diameter of PSSP@ART-ISMN increased to 210 ± 20.35 nm at a GSH concentration of 10 mM (Fig. 1I, J). Taken together, these results confirmed that the PSSP@ART-ISMN was stable enough under simulated physiological conditions, but was rapidly released in the reducing environment.

### Cellular uptake and selective toxicity of PSSP@ART-ISMN in vivo

**Fig. 2:**
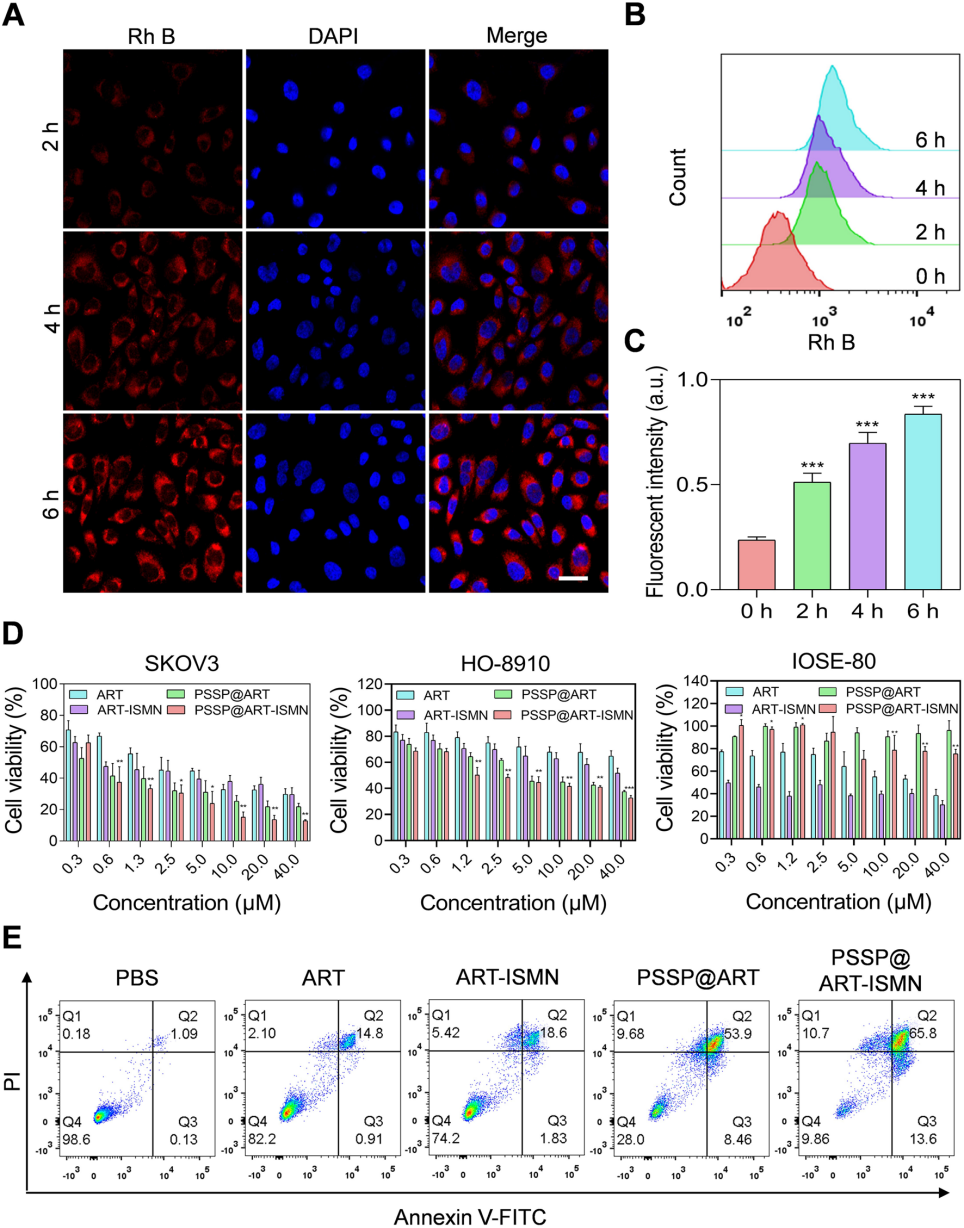
Cell internalization of PSSP@ART-ISMN and its cytotoxicity in vitro. **A** CLSM images of SKOV3 cells treated with PSSP@Rh B for 2, 4, and 6 h, scale bar = 25 µm. Flow cytometry (**B**) and semi-quantitative analysis (**C**) of SKOV3 cells after incubation with PSSP@Rh B for 2, 4, and 6 h. **D** Relative cell viability of SKOV3, HO8910, and IOSE-80 cells after treatment with ART, ART-ISMN, PSSP@ART, and PSSP@ART-ISMN for 72 h. Statistical analyses were carried out between free ART group and PSSP@ART-ISMN NPs group. **E** SKOV3 cell apoptosis images after different treatments for 24 h. *P < 0.05, **P < 0.01, and ***P < 0.001; ns indicates *P* > 0.05

Intracellular uptake is a key process for controlling drug bioavailability in vivo [32]. The fluorescent dye Rh B was encapsulated into PSSP@ART-ISMN (PSSP@Rh B) and detected under a confocal laser scanning microscope (CLSM). The increasing red fluorescence intensity was observed as a function of time from 2 to 6 h, indicating a gradual internalization of PSSP@ART-ISMN in the cells (Fig. 2A). Subsequently, flow cytometry analysis was performed to monitor the internalization processes of PSSP@ART-ISMN (Fig. 2B). Consistently, the uptake of SKOV3 cells increased nearly four-fold after a 6-h incubation (Fig. 2C), indicating the effective intracellular uptake of PSSP@ART-ISMN.

GSH concentration is much higher at the tumor sites due to the abnormal cellular metabolism [33]. The outstanding stability of PSSP@ART-ISMN ensures that it can maintain its structural stability and integrity during blood circulation until a GSH trigger is applied at the tumor site. Free ISMN produces NO, which is not toxic to cells (Additional file 1: Figure S3), but with the synergistic effect of ART, dramatic tumor-killing effects of PSSP@ART-ISMN were observed in cancer cells. In addition, no significant toxicity was observed in PSSP@ART-ISMN treated normal cells (Fig. 2D). This may be due to the high concentration of intracellular GSH in tumor cells, which triggers the release of PSSP@ART-ISMN. The in vitro tumor cell killing effects were also confirmed by cell apoptosis evaluation (Fig. 2E, Additional file 1: Figure S4). PSSP@ART-ISMN (79.40%) significantly increased cell apoptosis compared to free ART (15.71%). These results demonstrated that PSSP@ART-ISMN had a significant killing effect on tumor cells and excellent biosafety in normal cells.

### In vitro antitumor mechanisms of PSSP@ART-ISMN

**Fig. 3:**
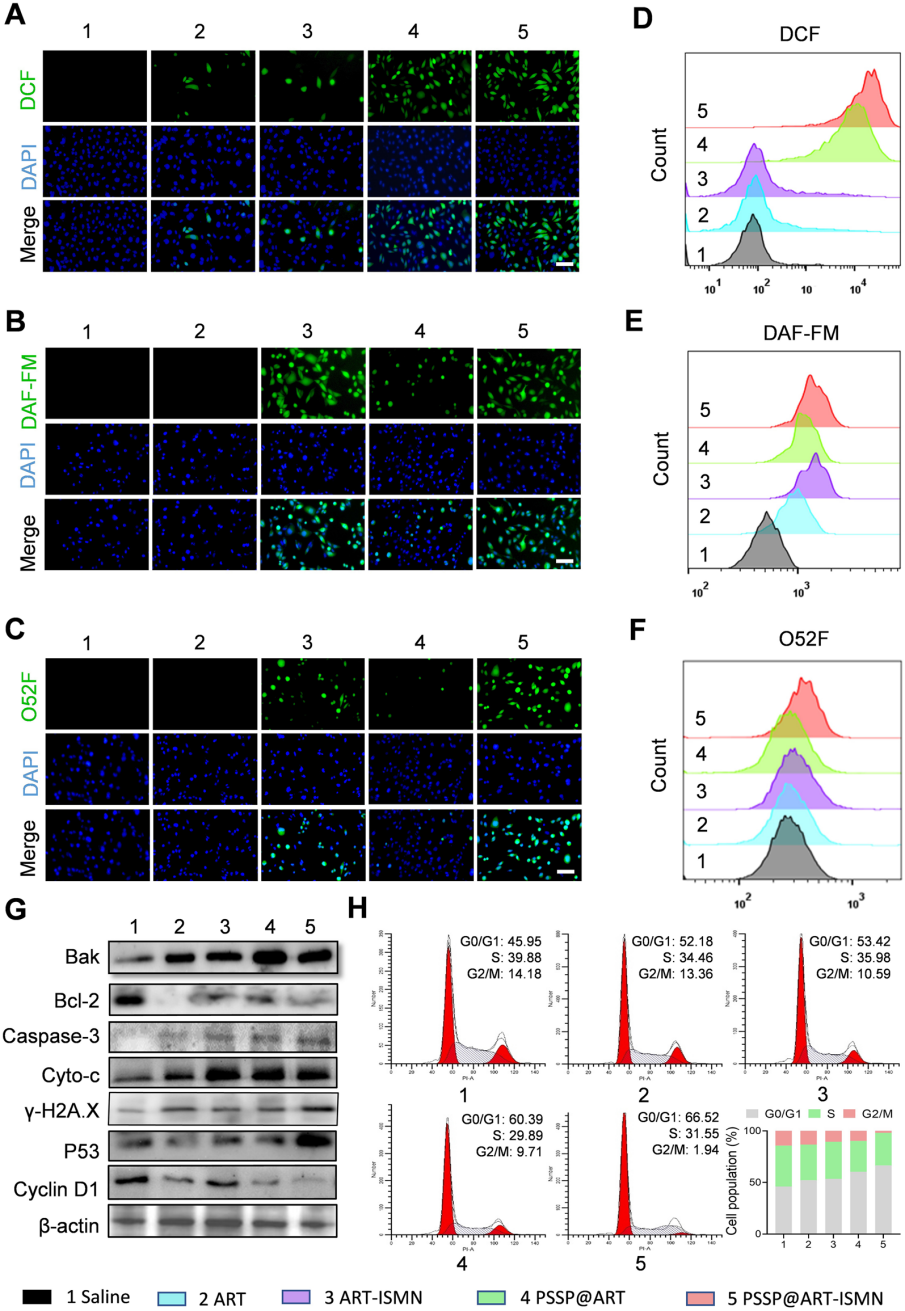
Anticancer mechanism of PSSP@ART-ISMN. **A**–**C** Intracellular ROS, NO, and RNS levels were detected by DCFH-DA, DAF-FM-DA, and O52D bio-probes, respectively, scale bar = 50 µm. **D**–**F** Flow cytometry analysis of intracellular ROS, NO, and RNS generation. **G** Western blot analysis of proteins related to mitochondria-dependent apoptosis, DNA damage, and cell cycle arrest. **H** Cell cycle study and quantitative analysis of SKOV3 cells after various treatments

ROS are cellular metabolites that widely exist in living organisms [34, 35]. A low concentration of ROS is necessary for the growth and development of living organisms, while a high concentration of ROS is known to cause oxidative damage to cellular organelles, such as lysosomes and mitochondria [36]. ART exerts an antitumor effect by increasing ROS within the tumor microenvironment. The intracellular ROS levels in SKOV3 cells were explored using a DCFH-DA fluorescence probe. The green fluorescence intensity in the PSSP@ART-ISMN and PSSP@ART treated groups was higher than that in other groups (Fig. 3A, Additional file 1: Figure S6), suggesting that PSSP@ART-ISMN and PSSP@ART markedly increased the intracellular level of ROS, which was also confirmed by flow cytometry (Fig. 3D). The production of excess ROS leads to a dysregulated redox balance status, which can ultimately lead to mitochondrial dysfunction [37]. Protein expression in mitochondria-mediated apoptosis was estimated using western blotting. The anti-apoptosis protein Bcl-2 was inhibited by PSSP@ART-ISMN, while the apoptosis proteins Bak, Caspase 3, and Cytochrome c were largely promoted by PSSP@ART-ISMN (Fig. 3G). These results demonstrated that the mitochondrial dysfunction induced by PSSP@ART-ISMN led to mitochondria-dependent apoptosis in SKOV3 cells.

On the other hand, excess ROS reacts with NO to generate highly toxic RNS, further enhancing the therapeutic effects [30, 38]. The intracellular NO and RNS levels were investigated after treatments with each agent. Green fluorescence was detected by CLSM and flow cytometry after incubation with the NO bio-probe (DAF-FM DA). The results showed that the free ART almost did not affect the intracellular NO level, while ART-ISMN and PSSP@ART-ISMN significantly enhanced the intracellular levels of NO (Fig. 3B, E, Additional file 1: Figure S6). Intracellular RNS accumulation was evaluated by fluorescent microscopy using an O52D fluorescent probe. SKOV3 cells incubated with ART and PSSP@ART did not produce RNS without the participation of NO, while green fluorescence was observed after treatment with ART-ISMN and PSSP@ART-ISMN, indicating the generation of RNS due to the simultaneous intracellular production of NO and ROS (Fig. 3C). Flow cytometry analysis also showed similar results (Fig. 3F). RNS directly caused DNA base lesions or indirectly facilitated DNA damage by hindering DNA replication progression. PSSP@ART-ISMN-treated SKOV3 cells showed a remarkable expression of γ-H2A.X, which is a marker of DNA damage response [39, 40], which further confirmed the DNA double-strand break caused by PSSP@ART-ISMN (Fig. 3G, Additional file 1: Figure S9).

Previous studies have reported that ART can regulate cancer growth by affecting cell cycle progression [7]. Therefore, the effect of PSSP@ART-ISMN on cell cycle distribution was detected by PI staining followed by flow cytometry. Cells in the PBS group were primarily in the G0/G1 phase (~ 45.95%), while up to 14.18% of cells were in the G2/M phase. After incubation with ART, ART-ISMN, PSSP@ART, and PSSP@ART-ISMN for 24 h, the population of SKOV3 cells in the G0/G1 phase increased to 52.18%, 53.42%, 60.39%, and 66.52%, respectively. The proportion of cells in the G2/M phase was significantly decreased to 1.94% in the PSSP@ART-ISMN group (Fig. 3H), indicating that PSSP@ART-ISMN prevented the cells from continuous proliferation and inducing cell death. Western blotting of cell cycle-related protein was used to analyze the expression after treatment with different agents. The expression of P53, a protein that promotes cell cycle arrest at the G1 checkpoint by transcriptionally activating several cell cycle regulatory factors, was significantly up-regulated due to the presence of ROS. In addition, a significant down-regulation of Cyclin D1 was also detected in the PSSP@ART-ISMN group (Fig. 3G, Additional file 1: Figure S9), indicating that cells were arrested in the G0/G1 phase. To conclude, these results suggested that ART released from PSSP@ART-ISMN induced cell cycle arrest in the G0/G1 phase, which led to cell apoptosis.

### In vivo antitumor efficiency and biocompatibility of PSSP@ART-ISMN

**Fig. 4:**
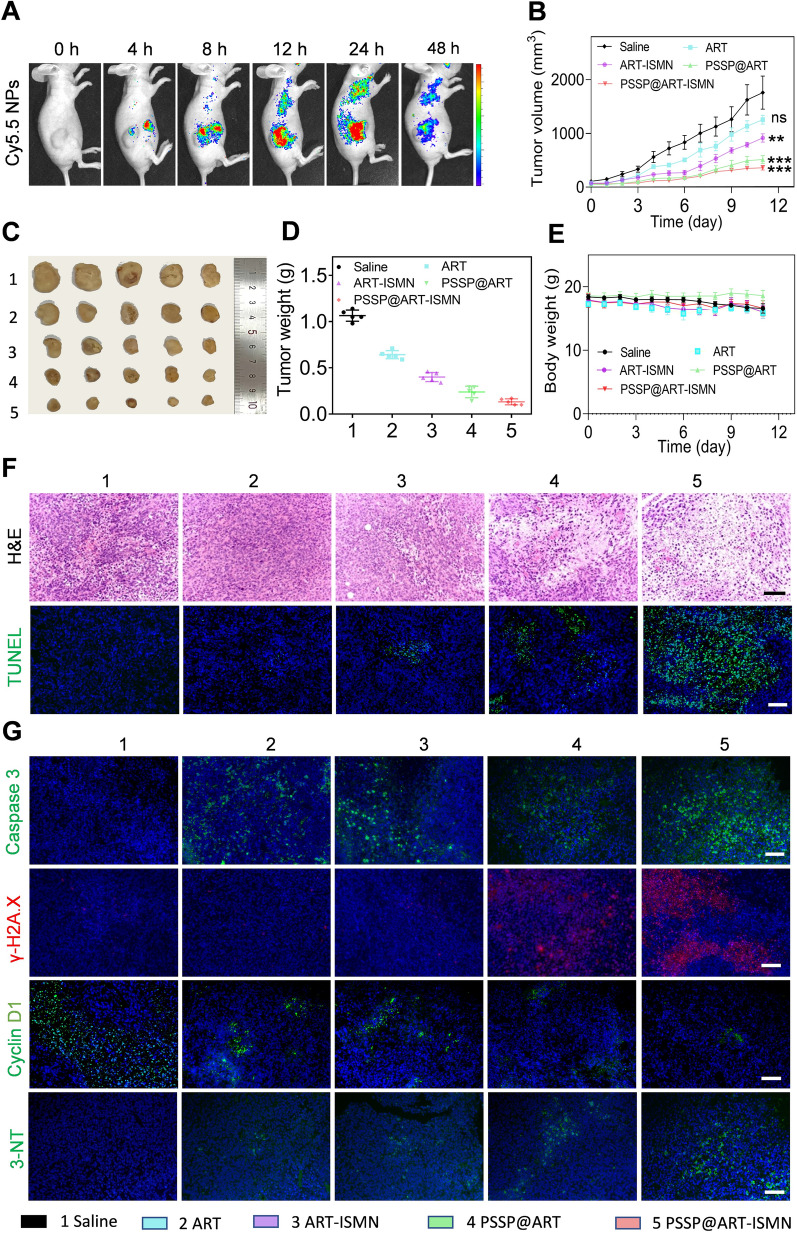
In vivo imaging, therapy, and biocompatibility of PSSP@ART-ISMN. **A** In vivo fluorescence biodistribution of Cy5.5-labeled PSSP@ART-ISMN in SKOV3 tumor-bearing mice. **B** Tumor growth curves of SKOV3 tumor-bearing mice receiving different therapies (n = 5). Representative photographs (**C**) and tumor weight (**D**) after treatment for 12 days. (**E**) Mouse body weight change curve over the duration of treatment. **F** H&E and TUNEL staining analysis of tumor sections, scale bar: 200 µm. **G** Immunofluorescence staining of tumor sections. *P < 0.05, **P < 0.01, and ***P < 0.001; ns indicates *P* > 0.05

Motivated by the in vitro antitumor effects, the PSSP@ART-ISMN antitumor effects in vivo were further investigated. To determine the targeting properties of PSSP@ART-ISMN in vivo, Cy5.5-labeled PSSP@ART-ISMN was injected into mice intravenously. As a result, the in vivo and ex vivo fluorescence signals accumulated at the tumor site, revealing an effective tumor enrichment ability of the NPs (Fig. 4A, Additional file 1: Figure S11).

Then, the SKOV3 tumor-bearing mouse model was established using the method described in the supporting information section. Once the tumor volume exceeded 50 mm^3^, tumor-bearing mice were randomized into saline, ART, ART-ISMN, PSSP@ART, and PSSP@ART-ISMN groups (n = 5). On days 1, 3, 5, 7, and 9, the mice were given an intravenous injection at an ART dose of 50 mg/kg, and tumor volumes were monitored every two days after drug administration. Compared to the saline group, ART, ART-ISMN, and PSSP@ART showed an antitumor effect, although PSSP@ART-ISMN was considered to be the best, which significantly inhibited the tumor volume to 360 mm^3^ (Fig. 4B). All mice were sacrificed after 11 days of treatment, and the tumors were isolated, photographed, and weighed. The tumor weight of mice treated with PSSP@ART-ISMN was only 0.13 ± 0.03 g, which was remarkably less than that in the saline (1.06 ± 0.06 g), ART (0.61 ± 0.17 g), ART-ISMN (0.40 ± 0.11 g), and PSSP@ART (0.24 ± 0.17 g) treatment groups, demonstrating the excellent antitumor effect of PSSP@ART-ISMN.

PSSP@ART-ISMN was more effective than other treatments, since more nuclear lysis and tumor necrosis were evident in H&E-stained tissues, which was also confirmed by the stronger apoptotic marker signal in TUNEL staining (Fig. 4F). In addition, the overexpression of Caspase 3 verified that the PSSP@ART-ISMN induced apoptosis via mitochondria-dependent pathway that was triggered by ROS. A dramatic increase in DNA damage indicator γ-H2A.X and a significant decline in the G0/G1 phase arrest indicator Cyclin D1 also confirmed the mechanism of antitumor effects in vivo (Fig. 4G). Additionally, 3-Nitrotyrosine (3-NT) is a nitrifying protein produced by RNS binding to intracellular biomolecules [41]. As expected, the highest 3-NT expression was detected in tumors of mice treated with PSSP@ART-ISMN (Fig. 4G, Additional file 1: Figure S10).

**Fig. 5:**
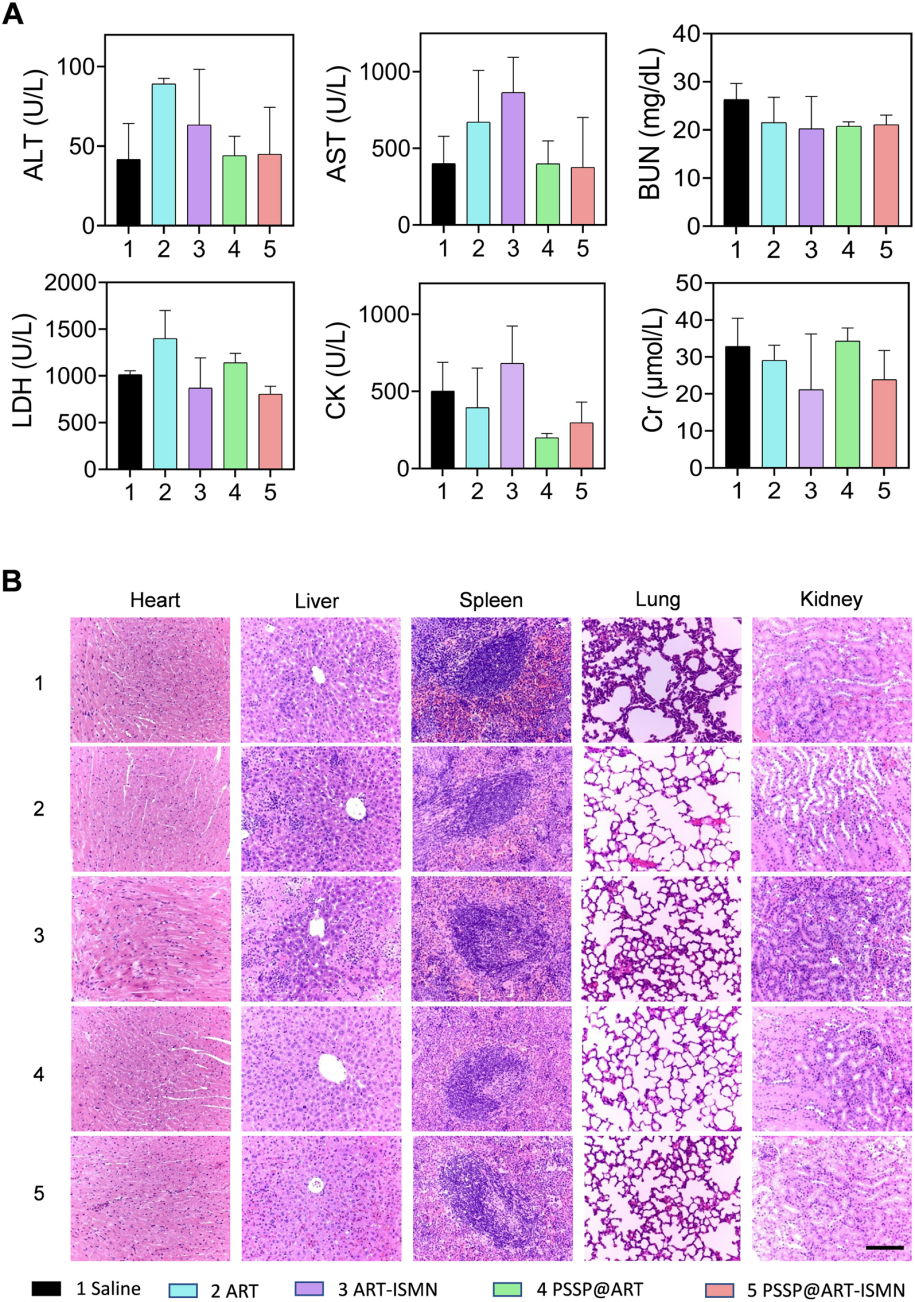
In vivo systemic toxicity study. **A** Evaluation of serum biochemical content in SKOV3 tumor-bearing mice after treatment with saline, ART, ART-ISMN, PSSP@ART, and PSSP@ART-ISMN (n = 3). **B** Histological analysis of main organs after treatment with different agents. Data are presented as mean ± SD, scale bar: 200 µm

SKOV3 tumor-bearing mice were sacrificed after treatment and blood samples were collected for blood biochemical analysis. The main organs and tumor tissues were harvested for H&E and immunofluorescence staining. All serum biochemical assays were within the reference ranges and there were no statistical differences among groups (Fig. 5A). No evident organ damage was noted in H&E-stained sections (Fig. 5B). No significant body weight loss occurred in either of the treatment groups (Fig. 4E), demonstrating that PSSP@ART-ISMN had good biocompatibility in vivo.

## Conclusion

In conclusion, a prodrug with nano-assembly strategy that co-delivers the NO and ROS donors was synthesized in the present study. The GSH responsiveness, selective cytotoxicity to tumor cells, preferable drug release capacity, favorable biocompatibility, and effective tumor-killing effect were characterized. The in vitro and in vivo results revealed that PSSP@ART-ISMN can (i) induce the production of ROS that contributes to mitochondrial damage; (ii) provide NO and ROS in the tumor cells, which further reacted to generate highly toxic RNS and caused DNA damage; and iii) arrest cell cycle at the G0/G1 phase and lead to apoptosis. Overall, this encouraging therapeutic outcome offers a creative co-delivery strategy for ovarian cancer therapy.

## Supplementary Information

**Additional file 1.** Additional information.

## Data Availability

All data generated or analyzed during this study are included in this published article and the Additional Information.
