# Supplementary material for: Synergetic delivery of artesunate and isosorbide 5-mononitrate with reduction-sensitive polymer nanoparticles for ovarian cancer chemotherapy
Source: J Nanobiotechnology. 2022 Nov 5;20:471. doi: 10.1186/s12951-022-01676-3 (PMC9636721; doi:10.1186/s12951-022-01676-3)
Supplement: Supplementary file 1 — Additional file 1. Additional information. [file 12951_2022_1676_MOESM1_ESM.docx]

**Additional information**

**Synergetic Delivery of Artesunate and Isosorbide 5-Mononitrate with Reduction-Sensitive Polymer Nanoparticles for Ovarian Cancer Chemotherapy**

^a^ Department of Obstetrics and Gynecology, The Third Affiliated Hospital of Southern Medical University, Guangzhou 510630, China

^b^ Southern Medical University Shenzhen Stomatology Hospital (Pingshan), Shenzhen 518000, China

^c^ Shenzhen Longhua District Central Hospital, Shenzhen 518110. China

^d^ Zhujiang Hospital of Southern Medical University, Guangzhou 510280, China

^e^ School of Pharmaceutical Sciences, Guangdong Provincial Key Laboratory of New Drug Screening, Southern Medical University, Guangzhou 510515, China

^f^ Department of Gynecology, Obstetrics and Gynecology Center, Zhujiang Hospital, Southern Medical University, Guangzhou 510280, China

^g^ Department of Laboratory Medicine, Dongguan Institute of Clinical Cancer Research, Affiliated Dongguan Hospital, Southern Medical University, Dongguan 523018, China

* Corresponding authors

E-mail: [13622893457@163.com(Y.-L.-Xiao)](mailto:13622893457@163.com(Y.-L.-Xiao)), [yuzq@smu.edu.cn](mailto:yuzq@smu.edu.cn) (Z.-Q. Yu), [douwangxuefeng@163.com](mailto:douwangxuefeng@163.com) (X.-F. wang)

^1^ These authors contributed equally.


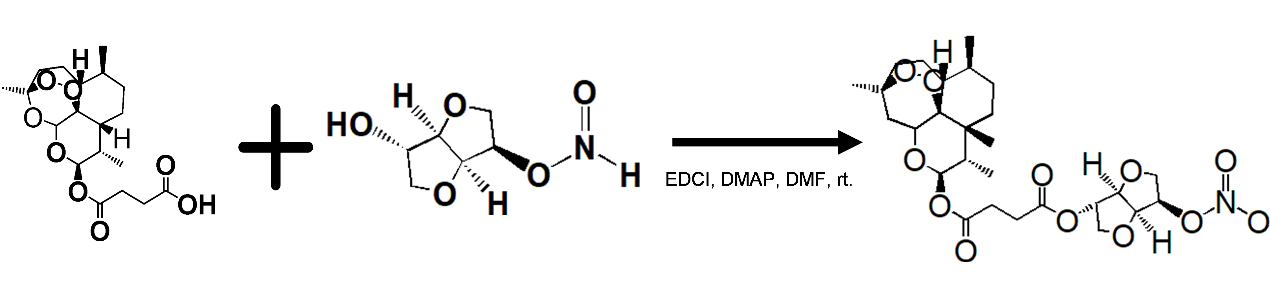


**Scheme S1.** Synthesis route of ART-ISMN.


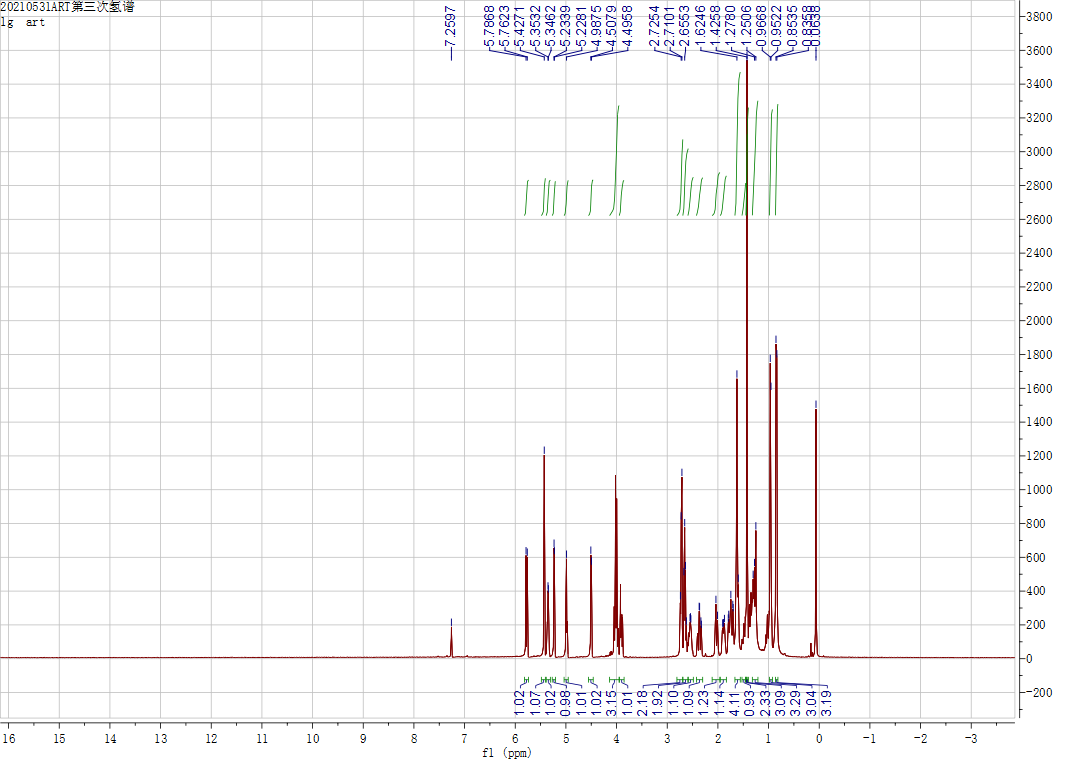

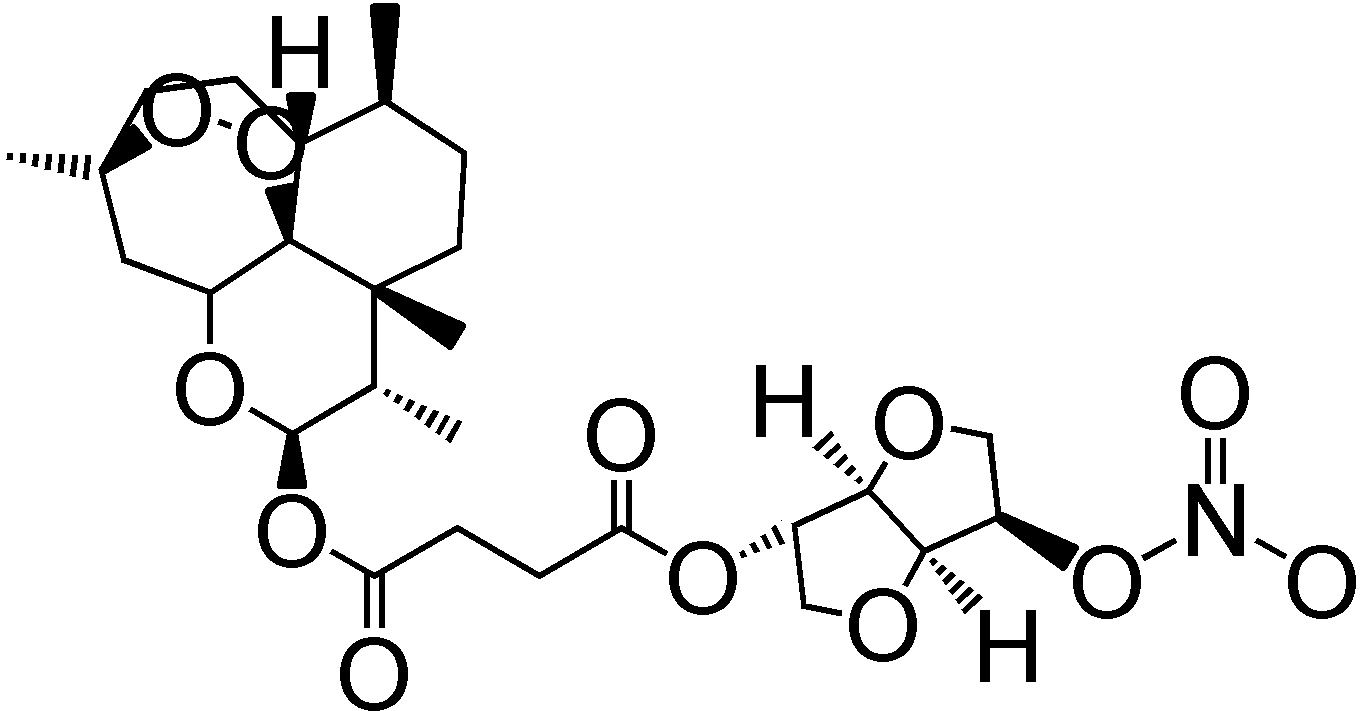


**Figure S1.** ^1^H NMR spectrum of ART-ISMN in DMSO-d6.


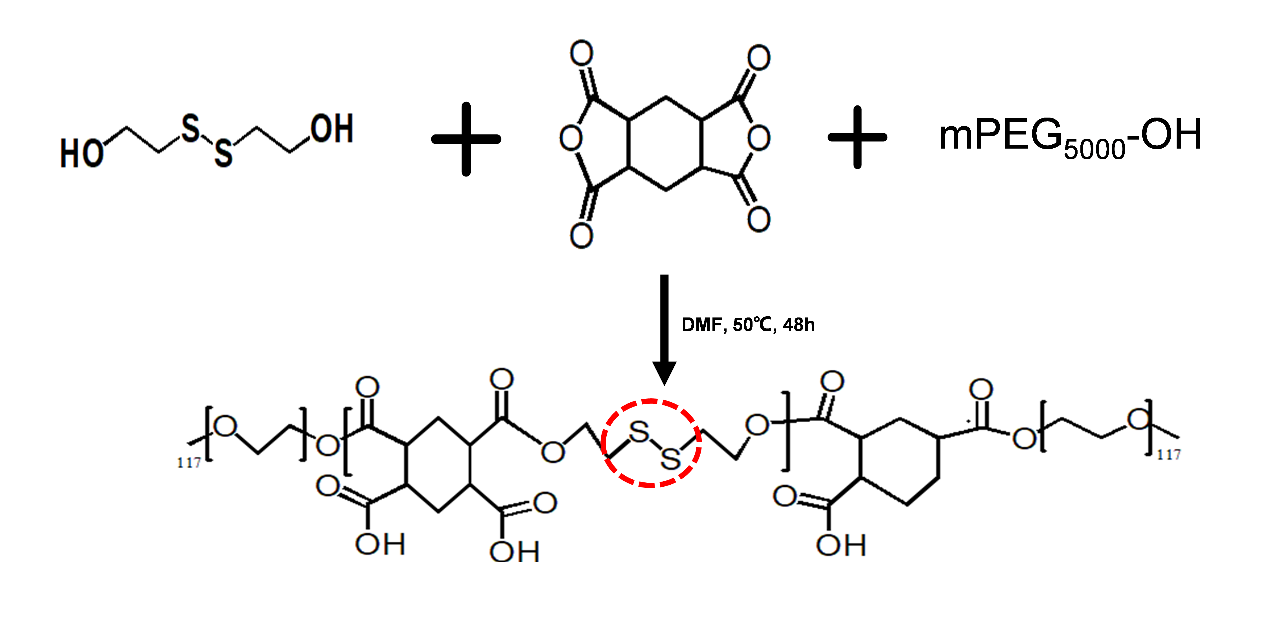


**Scheme S2.** Synthesis route of PSSP.


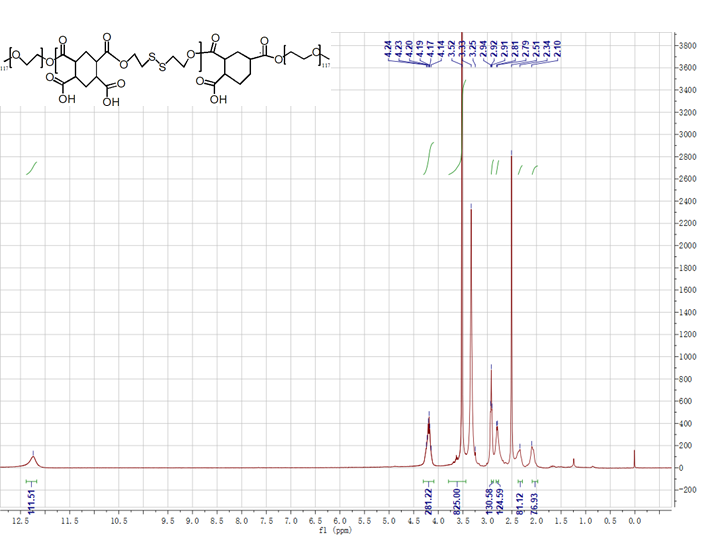


**Figure S2.** ^1^H NMR spectrum of PSSP in DMSO-d6.

| **ART-ISMN：PSSP**  **in feed** | **1:100** | **5:100** | **1:10** | **2:10** | **4:10** |
| --- | --- | --- | --- | --- | --- |
| Size (nm) | 163.00 | 174.20 | 165.30 | 158.23 | 227.00 |
| PDI | 0.19 | 0.16 | 0.25 | 0.16 | 0.19 |
| Zeta potential (mV) | -1.80 | -21.83 | -18.70 | -24.97 | -26.67 |
| Drug loading rate (%) | 0.48±0.03 | 0.82±0.02 | 1.88±0.08 | 3.85±0.10 | 3.85±0.05 |

**Table S1.** Size, PDI, zeta potential and drug loading rate in the obtained PSSP@ART-ISMN under different feed ratios of PSSP and ART-ISMN.


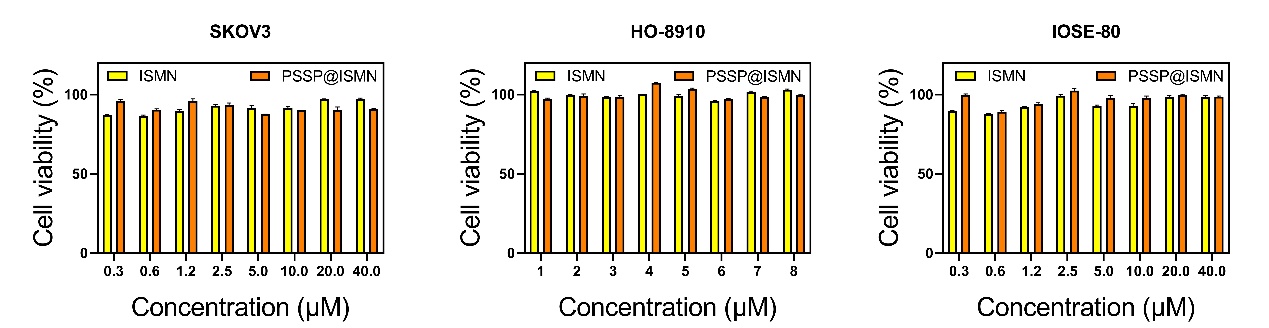


**Figure S3**. Relative cell viability of SKOV3, HO8910, and IOSE-80 cells after treatment with ISMN and PSSP@ISMN for 72 h.


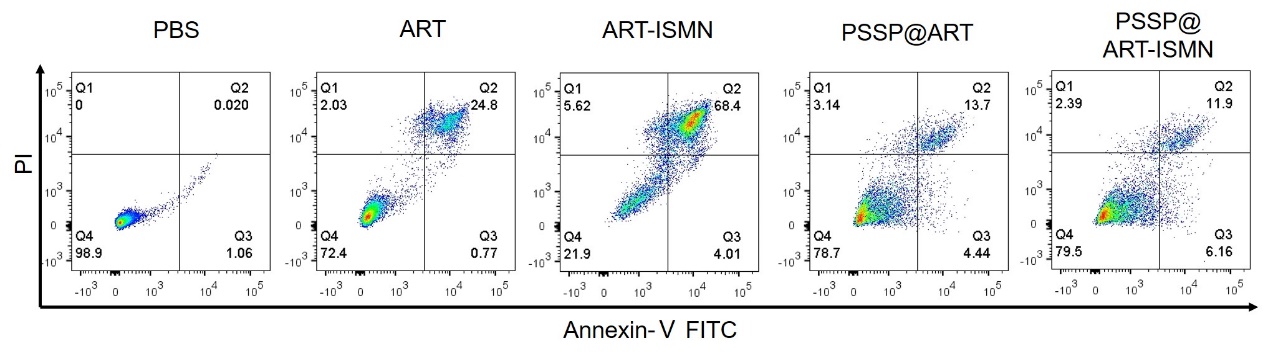


**Figure S4.** IOSE-80 cell apoptosis images after different treatments for 24 h.


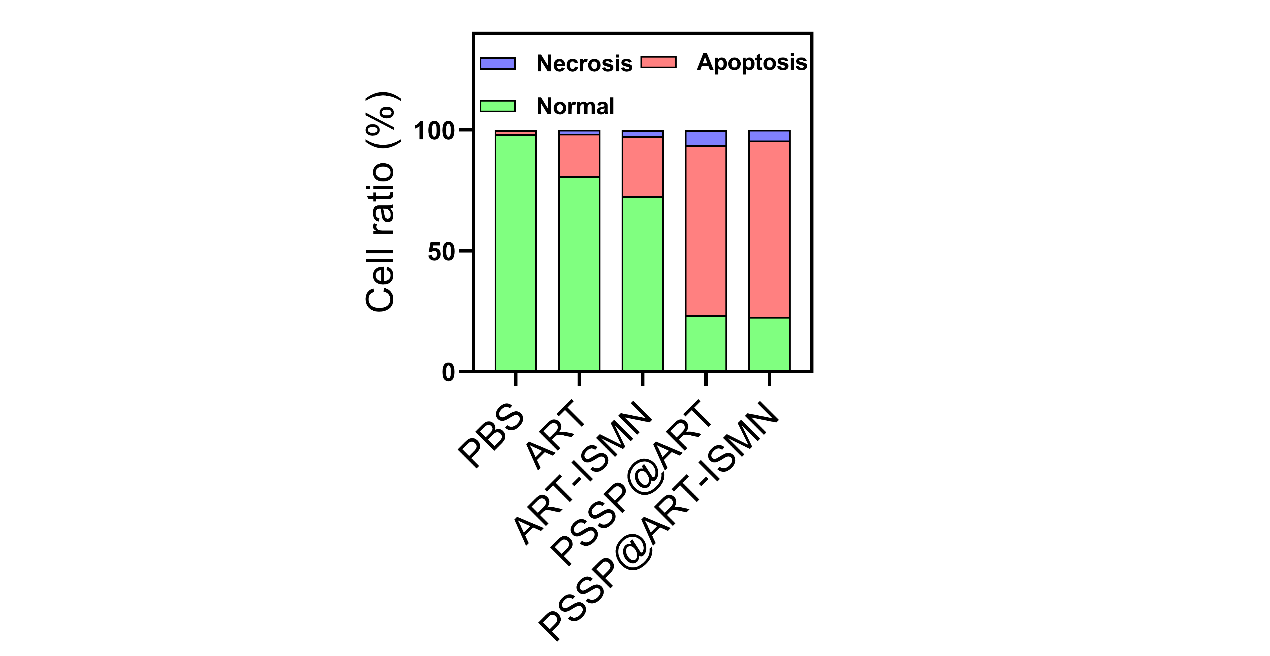


**Figure S5.** Statistical analysis of apoptosis.


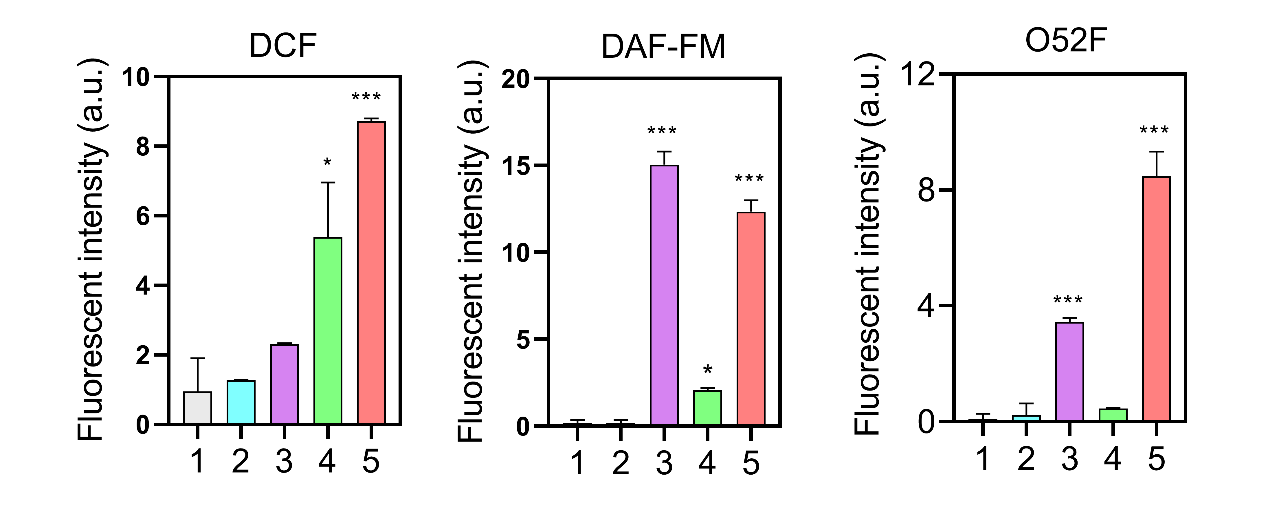


**Figure S6.** The semi-quantitative analysis of intracellular NO, ROS, and RNS levels detected by DCFH-DA bio-probe, DAF-FM-DA bio-probe, and O52D bio-probe. **P* < 0.05, ***P* < 0.01, ****P* < 0.001.


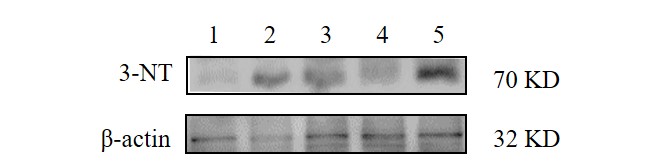


**Figure S7.** Western blot analysis of 3-NT


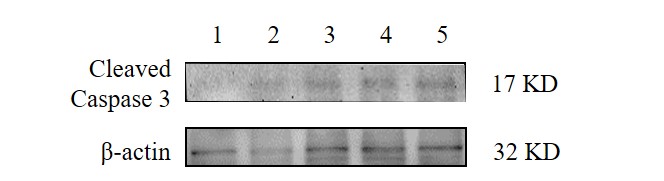


**Figure S8.** Western blot analysis of Cleaved Caspase 3


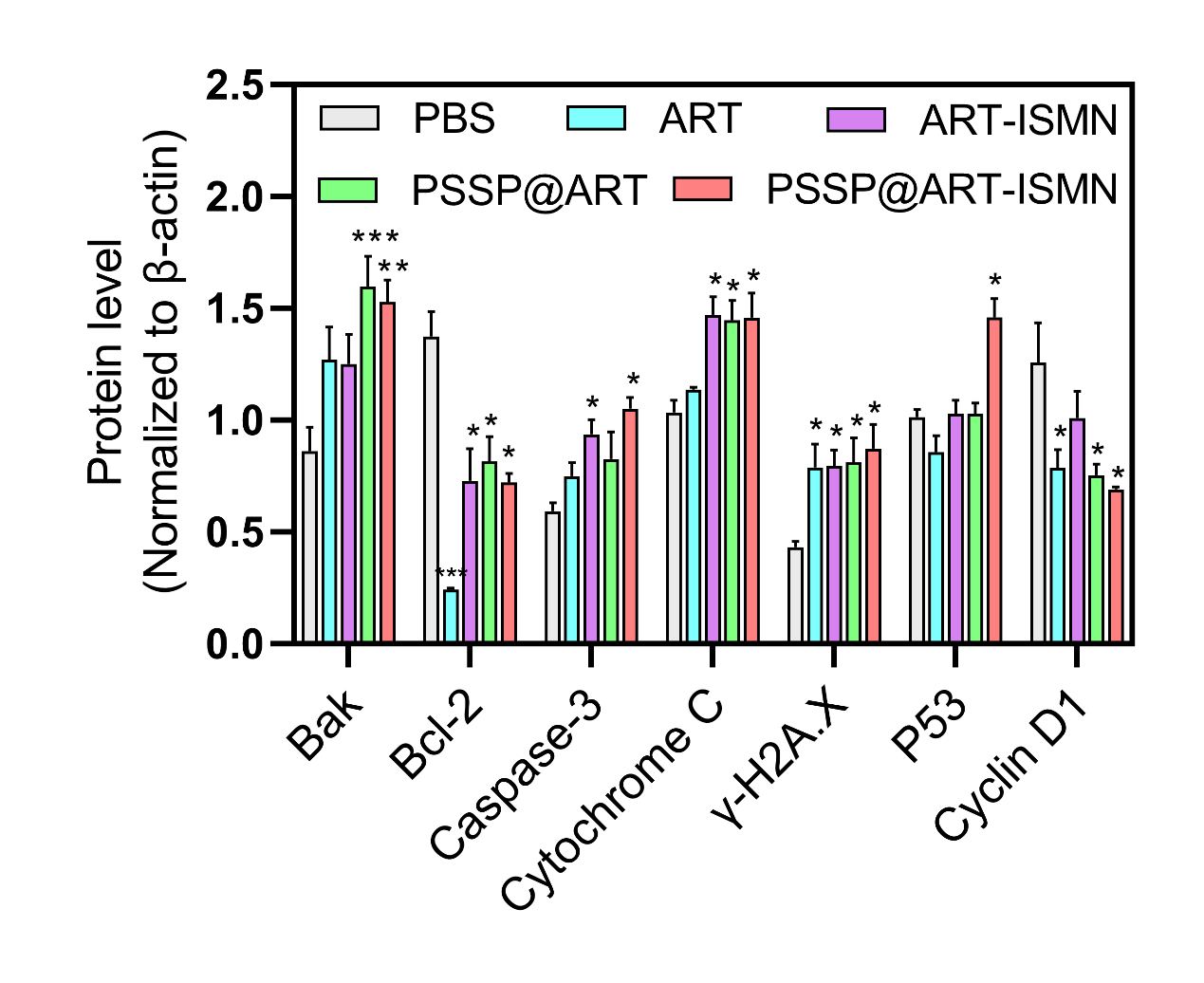


**Figure S9**. Expression levels of indicated proteins in Skov3 cells were detected using semiquantitative analyses. β-actin was used as a control. **P* < 0.05, ***P* < 0.01, ****P* < 0.001.


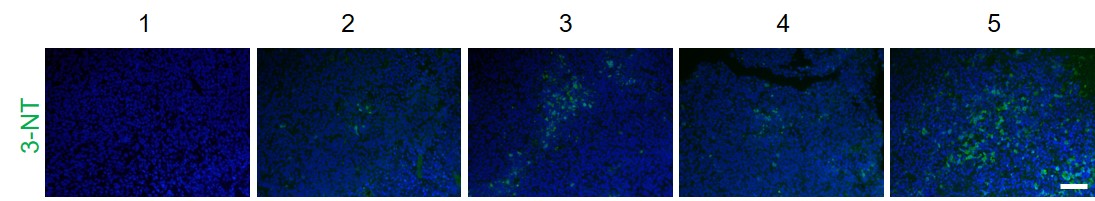


**Figure S10**. Immunofluorescence staining of 3-NT


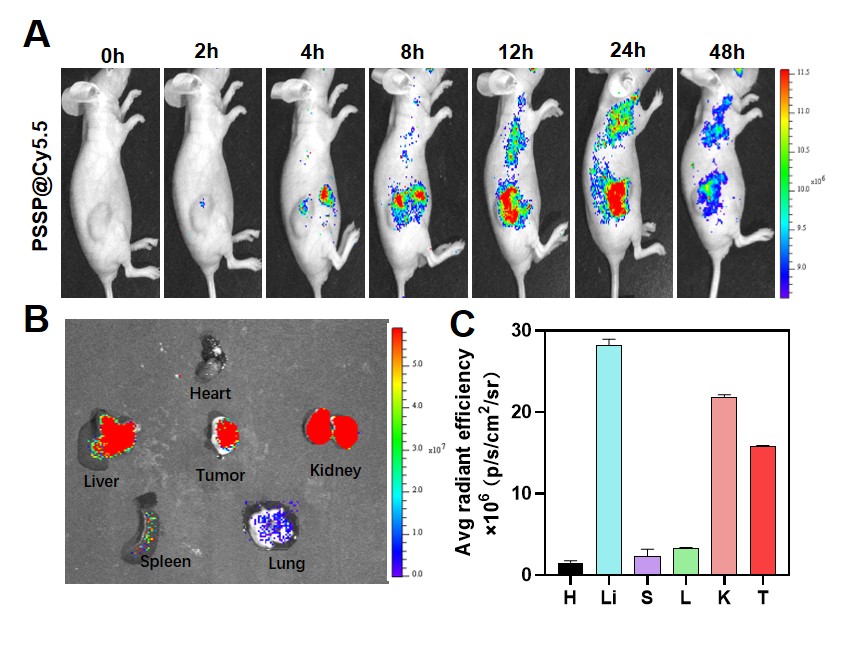


**Figure S11.** (**A**) *In vivo* biodistribution of Cy5.5 labeled PSSP@ART-ISMN in SKOV3 tumor bearing BALB/c nude mice. Fluorescence images (**B**) and its corresponding quantification (**C**) of excised tumors and organs 24 h after the intravenous injection.
